# Pulmonary Manifestations at Different Stages in the Chronic Kidney Disease: An Observational Study

**DOI:** 10.7759/cureus.39235

**Published:** 2023-05-19

**Authors:** Anbumaran Parivakkam Mani, Shanmugapriya K, Raja Sundar, Sankalp Yadav

**Affiliations:** 1 Respiratory Medicine, Saveetha Medical College and Hospital, Chennai, IND; 2 Respiratory Medicine, Sri Lalithambigai Medical College and Hospital, Dr. MGR Educational and Research Institute, Tamil Nadu, IND; 3 Respiratory Medicine, Saveetha Medical Collage and Hospital, Tamil Nadu, IND; 4 Medicine, Shri Madan Lal Khurana Chest Clinic, New Delhi, IND

**Keywords:** sleep apnea, pleural calcification, pulmonary tuberculosis, pulmonary edema, chronic kidney disease (ckd)

## Abstract

Introduction: By a variety of pathogenic pathways, kidney diseases can have a direct negative impact on the lungs and worsen the prognosis for those with chronic renal disease. Chronic kidney disease (CKD) is a public health concern throughout the world. The relationship between the kidneys and lungs is crucial for maintaining acid-base balance, fluid homeostasis, and blood pressure control. These patients have a higher prevalence of lung dysfunction regardless of the disease's stage, including sleep apnea syndrome, pulmonary hypertension, and chronic obstructive pulmonary disease (COPD). The chance of getting a pulmonary consequence increases with the severity of kidney disease. In individuals with chronic renal disease, this study looked at the prevalence of several respiratory disorders.

Materials and methods: From February 2021 to October 2021, 70 CKD patients who were receiving care at the Saveetha Medical College and Hospital were taken into consideration for the study. Clinical assessment and pertinent tests, such as a pulmonary function test, chest radiography, CT chest, sputum analysis, and pleural fluid analysis were performed. To evaluate left ventricular function, echocardiography was performed. Selected patients underwent polysomnography.

Results: The study's population had a mean age of 50 years. There was a 20:50 sex ratio (M:F). Seventy percent of them had respiratory conditions, the most frequent of which was pleural effusion (70%), followed by pulmonary edema (52%). The pleural effusion was primarily transudative and right sided. Both tuberculous pleural effusion and pulmonary tuberculosis were detected in 2% of the population. Seven percent of them developed pneumonia. 10% of patients had thickening of the pleura. Using chest CT and x-ray, 3% of patients had pulmonary calcification visible. In 12 (60%) out of the 20 patients who were studied, sleep apnea was observed. Two patients with tuberculosis and pneumonia lacked the typical signs.

Conclusions: In our research study, CKD patients have a much higher preponderance of respiratory illnesses, which has negative effects on patient care.

## Introduction

Chronic kidney disease (CKD) is a significant public health issue. Patients with CKD at all disease stages experience several comorbidities as a result of the disease. According to estimates, between 11% and 13% population of the world is currently suffering from it. Renal disease frequently involves many organ dysfunctions, some of which are brought on by close contact with other organs and tissues. In both health and disease, the kidneys' and the lungs' functions are intertwined. The interplay of the kidneys and lungs directly affects the maintenance of acid-base balance, blood pressure control, and fluid homeostasis. Maintenance dialysis can only partially replace kidney function [1]. The majority of patients currently receiving one of the three treatment modalities-hemodialysis (HD), peritoneal dialysis, or kidney transplant-are on HD [2]. A connection between HD and pulmonary hypertension was recently found (PH) [3].

The coaction of the kidneys and lungs has a direct impact on a number of important physiological processes. The precise mechanisms underlying a few of these are unknown, but some of these include changes in volume status, variations in plasma oncotic pressure, hypertension with diastolic dysfunction, arteriovenous fistulas (AVF), anemia, uremic lung, volume overload with interstitial pulmonary emphysema, high cardiac output state, changes in bone and mineral metabolism, concurrent heart failure, and altered immune function [4,5]. Uremic endothelial dysfunction makes PH worse by upsetting the balance between vasodilators (such as prostacyclins and nitric oxide) and vasoconstrictors (like endothelin 1 and plasma asymmetric dimethylarginine and thromboxane) [6,7]. Other hypothesized causes include extra-osseous vascular calcification, HD tubing or dialyzer microbubbles, and recurrent pulmonary thromboembolic disease [8-10].

Higher death rates are linked to PH in end-stage renal disease (ESRD). Patients on hemodialysis with PH had a mortality rate of 30.4%, in comparison to 8.5% for those without PH, in observational research [11]. In carefully chosen situations, certain considerations like endothelin receptor antagonists and prostacyclin analogs may be taken into account. Lungs in CKD can be significantly weakened pathologically [2]. These people have a higher prevalence of lung dysfunction regardless of the disease's stage, including sleep apnea syndrome, pulmonary hypertension, and chronic obstructive pulmonary disease (COPD). The chance of getting a pulmonary consequence increases with the severity of kidney disease [12]. Moreover, due to persistent fluid overload, CKD patients frequently have a restrictive spirometry pattern [13]. Because of fluid retention, metabolic, endocrine, and cardiovascular issues, pulmonary edema, and respiratory muscle dysfunction increase in frequency as the glomerular filtration rate (GFR) declines [14]. There are few statistics on the incidence and prevalence of PH in CKD among patients in India. At our tertiary care facility, we concentrated on the pulmonary symptoms at various stages of CKD.

## Materials and methods

A hospital-based observational study was conducted from February 2021 to October 2021, among 70 patients with CKD and pleural effusion at the Saveetha Medical College and Hospital. Patients aged 18 years or more and those diagnosed with CKD based on KDIGO Clinical Practice Guidelines for the Evaluation and Management of CKD were enrolled in the study. Patients aged less than 18 years, patients with valvular heart disease, congenital heart disease, connective tissue disorders, and thyroid disorders, and patients with respiratory disease onset before the diagnosis of CKD were excluded from the study.

Patients' histories were taken, with special attention paid to CKD and respiratory illness symptoms, co-morbid diseases, CKD diagnosis time, and hemodialysis time. The focus of the clinical examination was on symptoms of CKD and respiratory illnesses. The following tests were performed on each subject: Chest CT scans, thoracic ultrasonography, chest x-rays, and renal function tests may be required. These tests included a pulmonary function test, sputum analysis, and pleural fluid analysis. A left ventricular function assessment using echocardiography was made. In certain circumstances, sleep research was carried out.

In terms of mean and standard deviation, the continuous variables were expressed. The numerical representation of the categorical measurements was used (percentages). SPSS version 26 (IBM, Chicago, IL, USA) was used for analysis after the data were gathered, inputted, and summarized in Microsoft Excel 2019. The mean (SD) was used to summarize continuous variables, and the proportion was used to summarize categorical variables like sex. The association between categorical variables was analyzed using the Chi-square test and a P-value <0.05 were considered statistically significant.

## Results

The demographic characteristics of the study population were tabulated in Table 1. The prevalence of various symptoms and signs in the study participants and the prevalence of various respiratory manifestations in the study participants were tabulated in Tables 2, 3, respectively. The investigation findings in pleural effusion and radiological findings among the study participants were mentioned in Tables 4, 5, respectively.

**Table 1:** Demographic characteristics of study participants CKD - Chronic kidney disease

Variables		Data
Mean age		50 years (Range: 18 – 65)
Sex ratio (M:F)		2.5:1
Co-morbidities	Hypertension	54
	Diabetes mellitus	34
Stages of CKD	Stage 1	11
	Stage 2	17
	Stage 3	9
	Stage 4	3
	Stage 5	30
Dialysis	Yes	54
	No	16

**Table 2:** Prevalence of various symptoms and signs in the study participants

Symptoms	Number of patients (n=70)
Dyspnea	60
Orthopnea	27
Fatigue	48
Pedal edema	52
Cough	48
Abdominal distension	44
Chest pain	24
Cyanosis	7
Fever	10
Hemoptysis	4
Hiccough	7
Pleuritic pain	4
Pleural rub	3
Paroxysmal nocturnal dyspnea	44

**Table 3:** Prevalence of various respiratory manifestations in the study participants

Respiratory disease	Number of patients (n=70)
Pulmonary edema	52
Pleural effusion	70
Pleuritis	22
Pulmonary tuberculosis	2
Pleural tuberculosis	2
Pneumonia	7

**Table 4:** Investigation findings in pleural effusion in the study participants

Investigation findings		Number of patients (n=70)
Grades of pleural effusion	Mild	31
	Moderate	27
	Massive	12
Unilateral effusion	Left	27
	Right	22
Bilateral effusion		21
Recurrent pleural effusion		8
Hemorrhagic effusion		2
Transdutative effusion		38
Exudative effusion		32
Elevated adenosine deaminase		16

**Table 5:** Radiological findings in the study participants

Radiological findings	Number of patients (n=70)
Pulmonary hypertension	20
Cardiomegaly	32
Pleural effusion	70
Pulmonary edema	52
Cavitary lesion	3
Pleural thickening	10
Pulmonary calcification	3
Consolidation	10
Pulmonary infiltrates	3
Interstitial lung disease	3

## Discussion

Regardless of its genesis, PH is a cumulative illness with rising mortality and morbidity that complicates heart, lung, or systemic ailments and is characterized by raised pulmonary artery pressure [15]. Recently, it was found that PH is a strong independent predictor of morbidity and mortality in HD patients [16,17].

Due to the high cost of treatment, CKD is a major public health concern worldwide. Notwithstanding the fact that people with CKD are more likely to die from cardiovascular diseases (CVDs) than from end-stage renal disease, the severity may have been overstated [18]. According to statistics from the Indian Registry, chronic glomerulonephritis (15.8%), hypertension (14.8%), and diabetic nephropathy (30.3%) are the most typical causes of CKD in India [19]. As the population ages and there are an increasing number of people with diabetes mellitus (DM), there is probably a parallel rise in the incidence of CKD. About 30% of DM patients develop diabetic nephropathy.

More than 800 million individuals globally, or more than 10% of the world's population, suffer from CKD, a degenerative illness [20]. Because they are least equipped to deal with its effects, low- and middle-income countries bear a disproportionately heavy cost from CKD. It is one of the few non-communicable illnesses whose death toll has increased in the past 20 years and has gotten to the top of the list of killers on a global scale. Clinical situations that include the lung and kidneys are quite complex and challenging. Due to its effects on vascular tone, acid-base balance, and fluid homeostasis, the physiological state of the lung is greatly impacted by CKD. Hemodynamic abnormalities in the lung are the root cause of modifications in ventilatory control, pulmonary congestion, capillary stress failure, and pulmonary vascular disease. Hemodynamic abnormalities in the kidney cause salt and water retention as well as a decline in renal function. Many illnesses that most frequently manifest as glomerulonephritis and alveolar bleeding impact both the lungs and the kidneys. Although respiratory care experts do occasionally see three of them - granulomatosis, Wegener's systemic lupus erythematosus, and Goodpasture's syndrome - the remainder of these conditions are uncommon or atypical [21]. Respiratory side effects of chronic renal failure include pulmonary edema, fibrinous pleuritis, lung calcification, and susceptibility to tuberculosis (TB). Urinothorax is a rare disease associated with obstructive uropathy. Patients with end-stage renal illness frequently experience sleep difficulties, with sleep apnea affecting 60% or more of these patients [22]. The effects of fluid overload, metabolic acidosis, and pulmonary edema frequently make it difficult to manage patients with acute renal failure. The following complication were noted during various stages of CKD.

Pulmonary edema

This is the most common complication among CKD patients with a high rate of morbidity and mortality. It is present in 52 patients and is the most common respiratory manifestation with dyspnea, paroxysmal nocturnal dyspnea, and erythema. Pulmonary edema in CKD patients is usually caused by left ventricular failure, which increases left ventricular filling pressure which leads to an increase in pulmonary hydrostatic pressure causing pulmonary edema. Left ventricular failure affects 30% to 70% of stage 4 CKD patients.

Pleural effusion

Reduced oncotic pressure and increased hydrostatic pressure can cause pleural effusion in CKD patients. Increased hydrostatic pressure is caused by hypervolemic salt retention. Thirty-eight patients showed a transudative type of effusion and 32 patients showed an exudative type of effusion. Out of 32 patients with exudative effusion, 16 patients were diagnosed with a TB effusion in major raised ADA levels.

Pleuritis

Twenty-two patients in our study had pleuritis, with 10 patients having pleural thickening and three patients having pleural calcification due to fibrinous inflammation of the parietal pleural in CKD patients elucidating long-standing pleural effusion in CKD patients.

Tuberculosis

TB is one of the most common emerging manifestations in CKD patients. In our study, 16 patients were diagnosed with TB effusion with an exudative type of effusion with raised ADA level, hitherto, TB was underdiagnosed and under concern in CKD patients. Now CKD is immuno- compromised states, and high detection of TB was elicited as in our study.

Pneumonia

*Streptococcus pneumoniae*, the most common pathogen, was identified as the cause of pneumonia in seven individuals in our study. Other than CKD, diabetes, hypertension, and CAD were also indirectly linked as cause of pneumonia in CKD patients.

The limitations of this study were the small sample size with most of the patients of CKD where on stage 5, and no long-term follow-up of the patients.

## Conclusions

In the upcoming years, it is expected that the prevalence of CKD and lung illness would rise dramatically globally, causing serious economic and societal impacts. A vital channel for controlling body homeostasis and avoiding the worsening of systemic illness is the kidney-lung axis. These organs have similar mechanisms that can contribute to the onset and development of both disorders. The results from this study show that pleural effusion, which typically manifests as dyspnea and pedal edema in CKD patients, is the most frequent respiratory manifestation. The complex mechanism of pleural effusion could be brought on by either an increase in hydrostatic pressure or a decrease in oncotic pressure. In CKD patients, TB must be suspected more aggressively. The small sample size of this study is a weakness since it may necessitate additional research to diagnose TB in CKD patients. To more accurately characterize effective therapy options, additional research into the interactions between these organs is required.
